# A Case of Kratom-induced Seizures

**DOI:** 10.7759/cureus.6588

**Published:** 2020-01-07

**Authors:** Hasnain Afzal, Michael Esang, Sabreen Rahman

**Affiliations:** 1 Psychiatry and Behavioral Sciences, Nassau University Medical Center, East Meadow, USA

**Keywords:** kratom, opioid, seizures, substance use

## Abstract

Kratom or Mitragna speciosa is a tropical tree that is indigenous to Southeast Asia, where it has been used for various medicinal reasons. In the West, it is used in the self-treatment of opioid withdrawal, pain, and a variety of mood and anxiety states. Two active ingredients in kratom are mitragynine and 7-hydroxymitragynine, which have affinity at the mu-opioid receptor among others. Kratom is easily available over the Internet and its use is increasing in the United States. It is currently listed by the Drug Enforcement Administration as a drug of concern. In 2017, the U.S. Food and Drug Administration started issuing a series of warnings about kratom, and by early 2018, it released a statement identifying 44 deaths related to kratom use. The Centers for Disease Control and Prevention has also reported 91 deaths directly linked to kratom use in 2019. Although its safety profile needs additional research for clarification, there have been reports of kratom-induced or kratom-related respiratory depression, hypothyroidism, secondary hypogonadism, hyperprolactinemia, psychosis, and seizures. We report a case of kratom-induced tonic-clonic seizures in a 27-year-old Caucasian male with a psychiatric history of anxiety, attention-deficit/hyperactivity disorder, benzodiazepine use disorder, and opioid use disorder. He was hospitalized after a witnessed tonic-clonic seizure. There was no significant metabolic abnormality on laboratory testing. Spinal cord and brain imaging were unremarkable, whereas his urine toxicology was positive for opioids only, which was likely a false-positive result due to cross-reactivity with his sleeping aids. He was evaluated by the Consultation-Liaison Psychiatry team for psychotic symptoms. On evaluation, the patient’s psychosis had resolved, but he endorsed racing thoughts, significant anxiety, and insomnia. He admitted to drinking three to four 8-mL bottles of Kratom daily for one-and-a-half years to self-medicate his anxiety after losing his health insurance. In the hospital, he was treated with anxiolytics, counseled to abstain from Kratom use, and was referred for substance use disorder treatment. This case highlights the life-threatening complications of Kratom that is easily available online.

## Introduction

Kratom or Mitragyna speciosa is a tropical tree that can grow as high as 16 meters and is native to Southeast Asia, the Philippines, and New Guinea [1]. Kratom, as it is known in Thailand, refers to various preparations derived from the tree and specifically from its leaves. Other names include Thom, Thang, Kakuam, Ketom, and Biak. Although reports of its use in these indigenous regions date back to the 1800s, it is now being cultivated in other parts of the world [2]. Traditional use involved chewing the fresh leaves or preparing tea from its dried leaves. Today, kratom is sold online and at a variety of retail outlets including bars, smoke shops, and even gas stations. It is sold as loosely chopped leaves, tablets, capsules, and concentrated extracts. Routes of use include inhalation and oral ingestion. Kratom is also added to cocktails, caffeinated beverages, cough syrup, and cannabinoid preparations for recreational use [2].

Over the years, there has been an increase in the use of kratom products in the United States (US) as a drug of abuse and self-treatment of opioid withdrawal and pain. Adverse effects associated with kratom use have caught the attention of federal regulatory bodies including the U.S. Food and Drug Administration (FDA) and the Drug Enforcement Administration (DEA), and these effects have included addiction, psychosis, seizures, liver injury, respiratory depression, coma, and even death. [3-7]. In 2016, the DEA proposed a temporary ban on kratom out of concern for public safety. This triggered negative reactions from advocacy groups, and, in 2017, the ban was rescinded. Individual states in the US, however, introduced legislative bills addressing kratom, with some states banning kratom products. Kratom is banned in the following states: Alabama, Arkansas, Indiana, Rhode Island, Vermont, and Wisconsin. Kratom is also banned in Washington D.C. Previously banned in Tennessee, the ban was reversed, making kratom legal for individuals 21 years or older, as of July 2018 [8].

In February 2018, the FDA released a statement on Kratom’s potential for abuse, addiction, and serious health consequences including death. The FDA also reported 44 deaths associated with Kratom use [9]. Efforts to advance regulation of kratom products have been driven by the risks posed by adulteration, which are extremely high concerning overdose and other adverse health consequences [10]. Krypton, a kratom product sold online is adulterated with an active metabolite of tramadol, O-desmethyltramadol, a substance that has been implicated in at least nine deaths [10].

In the US, analysts sponsored by the Centers for Disease Control and Prevention (CDC) in 32 states and the District of Columbia reviewed data on 27,338 overdose deaths that occurred from July 2016 to December 2017. They found that 152 of these decedents were positive for kratom on postmortem examination. Kratom was determined to be a cause of death for 91 of these 152 deaths. According to the CDC report published in April 2019, about 80% of the kratom-positive and kratom-involved deaths had a history of substance misuse [11]. Fentanyl and fentanyl analogs were the most frequently identified co-occurring substances in these deaths. Reports of other co-occurring substances in fatal overdoses have cited propylhexedrine (Datura stramonium), carisoprodol, morphine, and even seemingly innocuous agents such as diphenhydramine and caffeine [2,10,12]. The last two, however, have typically led to fatalities in combination with morphine. Considering that diphenhydramine is a CYP 2D6 inhibitor, one can understand how the toxicity risk of combination products containing diphenhydramine can be elevated [2].

Finally, in an unrelated case for the regulation of kratom and kratom products, the CDC linked kratom to a multistate outbreak of Salmonella infections in 2018. This outbreak involved 199 cases in 41 states and led to 50 hospitalizations, but no deaths were reported. No single source was identified, leading the CDC to advise that, in spite of a wide recall of these products, contaminated kratom products may still be in circulation [13].

This article presents a case of kratom-induced seizures in an individual with risk factors for kratom misuse. The aim is to raise awareness of the dangers of unregulated kratom use among individuals at risk of significant morbidity and mortality from such use. We hope that this report will contribute to the nuances in the ongoing conversations surrounding this controversial plant-based product.

## Case presentation

We report a case of kratom-induced tonic-clonic seizures in a 27-year-old Caucasian male with a psychiatric history of unspecified anxiety disorder, attention-deficit/hyperactivity disorder (ADHD), benzodiazepine use disorder, and opioid use disorder who presented with witnessed seizures. The patient went to smoke outside his house when his bother found him swinging his arm in the air "like catching a bug." He subsequently lost consciousness and started having a witnessed tonic-clonic seizure for which he was taken to the hospital and admitted for post-ictal confusion and medical workup. No epilepsy risk factors were reported. There were no significant metabolic abnormalities on laboratory testing. Spinal cord and brain imaging were equally unremarkable, whereas his urine toxicology was positive for opioids only. The latter was likely a false-positive result due to cross-reactivity with his over-the-counter sleeping aid, diphenhydramine. Diphenhydramine has been known to cause a false-positive opioid test on urine drug screen [14]. He strongly denied the use of opioids, admitted to drinking alcohol socially, and denied the use of benzodiazepines over the previous few years. He was evaluated by the Consultation-Liaison Psychiatry team for psychotic symptoms because he had been seen flaying his arms in the air prior to the seizure, in a manner reminiscent of someone attempting to catch bugs in the air. His brother had mentioned that there was nothing in the air around the patient when he had witnessed this behavior. On evaluation, however, the patient’s psychosis had resolved. He also denied any withdrawal symptoms but endorsed racing thoughts, significant anxiety, and insomnia. He admitted that he had been drinking up to three to four 8-mL bottles of kratom daily for one-and-a-half years to self-medicate his anxiety after losing his health insurance. He was diagnosed as having had a seizure secondary to kratom use. He was then treated with anxiolytics, counseled to abstain from kratom use, and was subsequently discharged after two days of inpatient stay with a referral for benzodiazepine use disorder and opioid use disorder treatment, as well as a follow-up appointment with a psychiatrist to continue managing the unspecified anxiety disorder.

## Discussion

**Figure 1:**
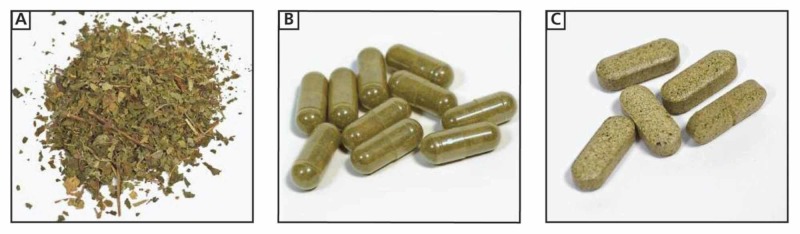
Images of kratom products that can be purchased at a “smoke shop” in the US: (A) chopped leaves, which are typically brewed into “kratom tea”, (B) capsules containing finely chopped leaves, and (C) compressed tablets containing leaves or resin.

The psychoactive properties of kratom, including its addictive potential, have been reported in the literature over the years. Figure 1 shows images of kratom products as they might appear at a typical purchase location [12]. 

When used at low dosages, it has stimulant effects that reduce fatigue and increase work capacity, akin to the effects of cocaine. The pharmacological mechanism responsible for this remains unclear. At high dosages, however, it has sedative-narcotic “morphine-like” properties and has therefore been used as an opium substitute. Table 1 summarizes the effects of kratom use at low and high doses, whereas Table 2 shows the effect of chronic kratom use, highlighting the potential presentation in withdrawal and dependence. 

**Table 1:** Effects of kratom use at low and high doses [12].

Effects of kratom at low doses (equivalent of 1-5 g of the raw leaves)	Effects of kratom at moderate to high doses (equivalent of 5-15 g of the raw leaves)
Increased work capacity, euphoria, alertness, sociability, heightened sexual desire, pupils are normal or very slightly contracted, blushing motor excitement, giddiness, loss of motor coordination (positive Romberg’s test), tremors of the extremities and face, anxiety, internal agitation (akin to akathisia) leading to dysphoria, aggressiveness, irritability	Analgesia, sedation, sweating, dizziness, nausea, dysphoria, euphoria, dreamlike state, miosis, constipation, itching

**Table 2:** Effect of chronic kratom use [12].

Effects of chronic kratom use
Weight loss
Constipation
Hyperpigmentation of the cheeks
Withdrawal syndrome and dependence: craving, weakness and lethargy, depression, anxiety, restlessness, hostility, aggression, emotional lability, rhinorrhea, lacrimation, dry mouth, myalgia, nausea, sweating, jerky movements of the limbs, tremors, sleep disturbances, psychosis with or without hallucinations (exacerbation or precipitation), higher suicide risk

**Figure 2:**
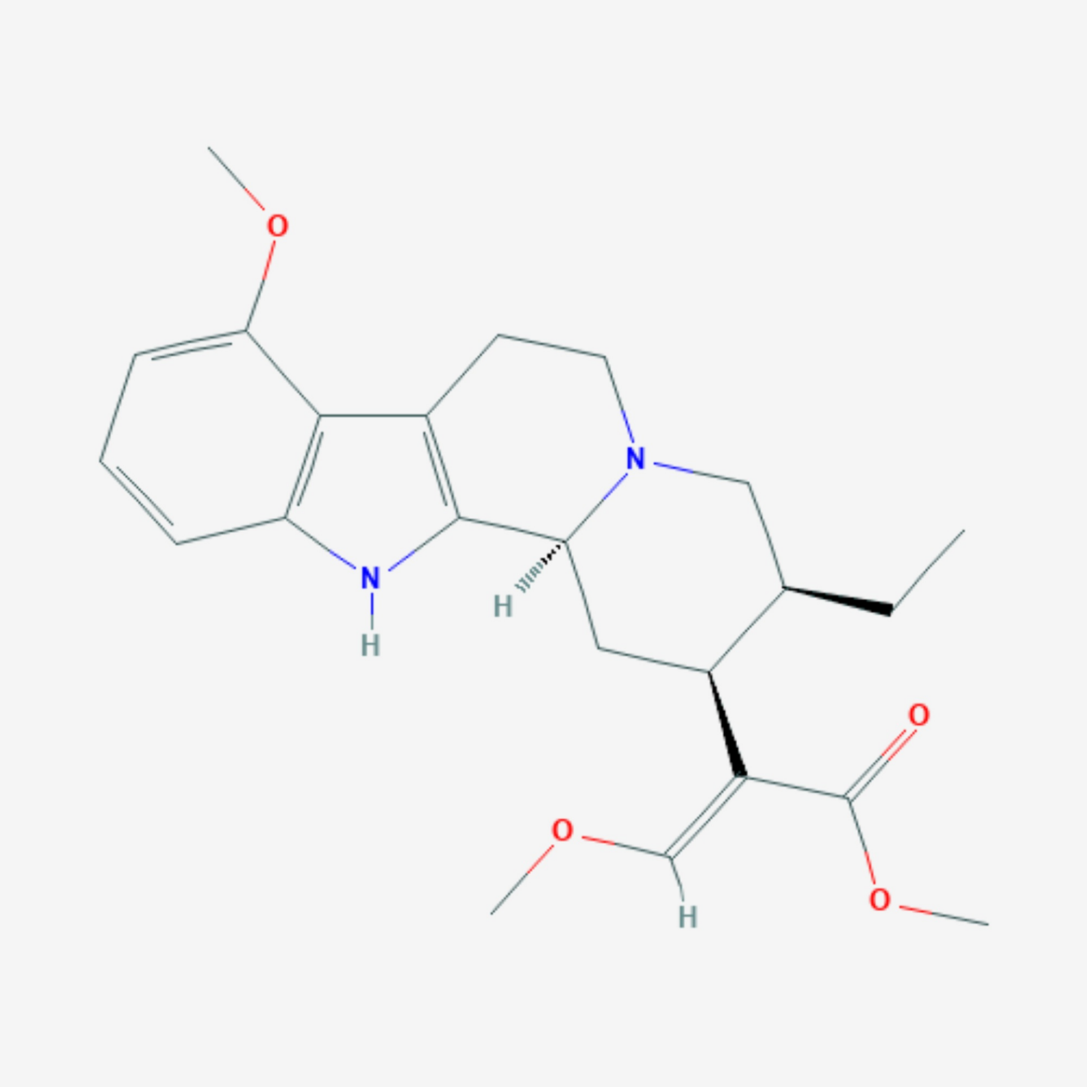
Mitragynine – molecular formula: C23H30N2O4; molecular weight: 398.50 g/mol.

**Figure 3:**
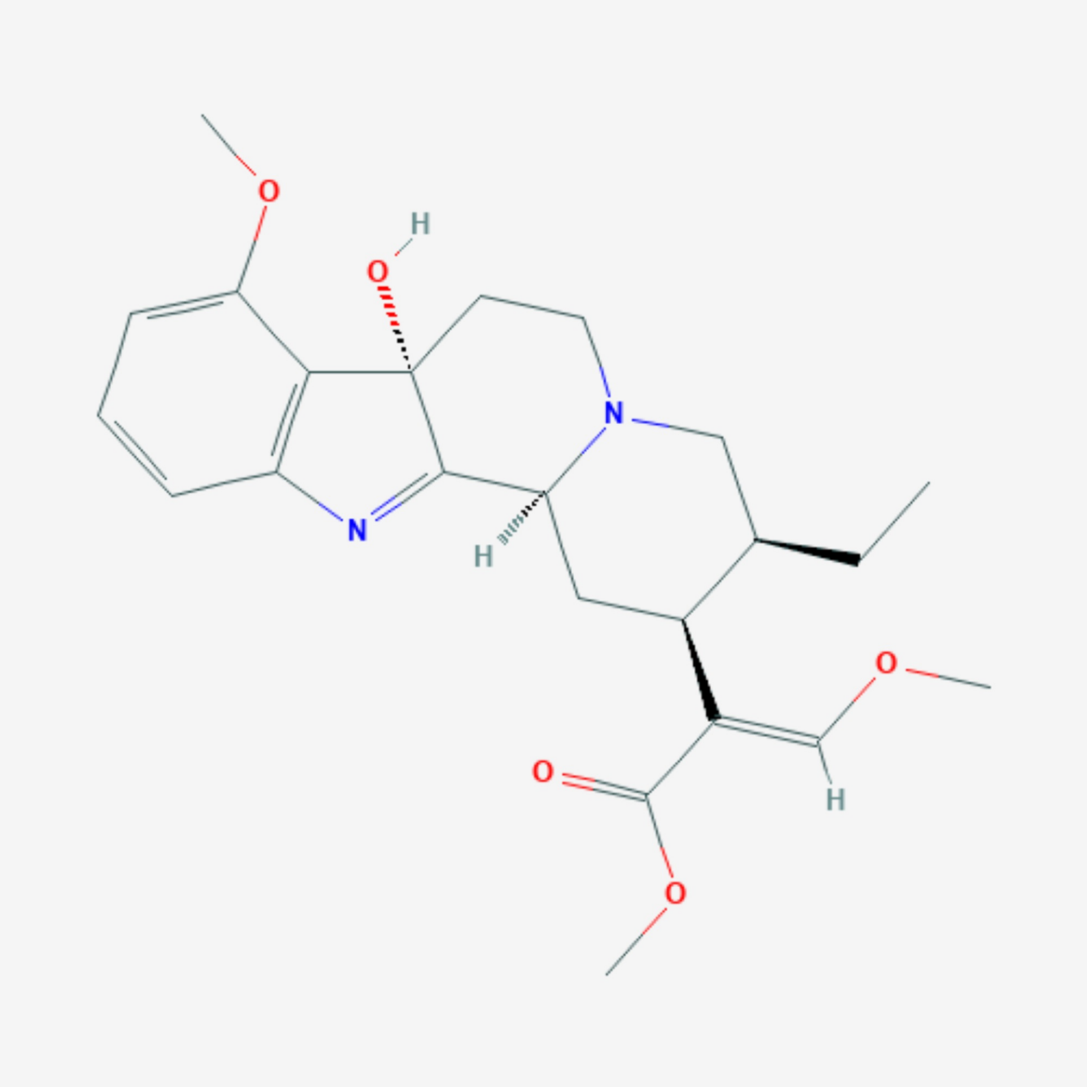
7-hydroxymitragynine – molecular formula: C23H30N2O5; molecular weight: 414.50 g/mol.

**Figure 4:**
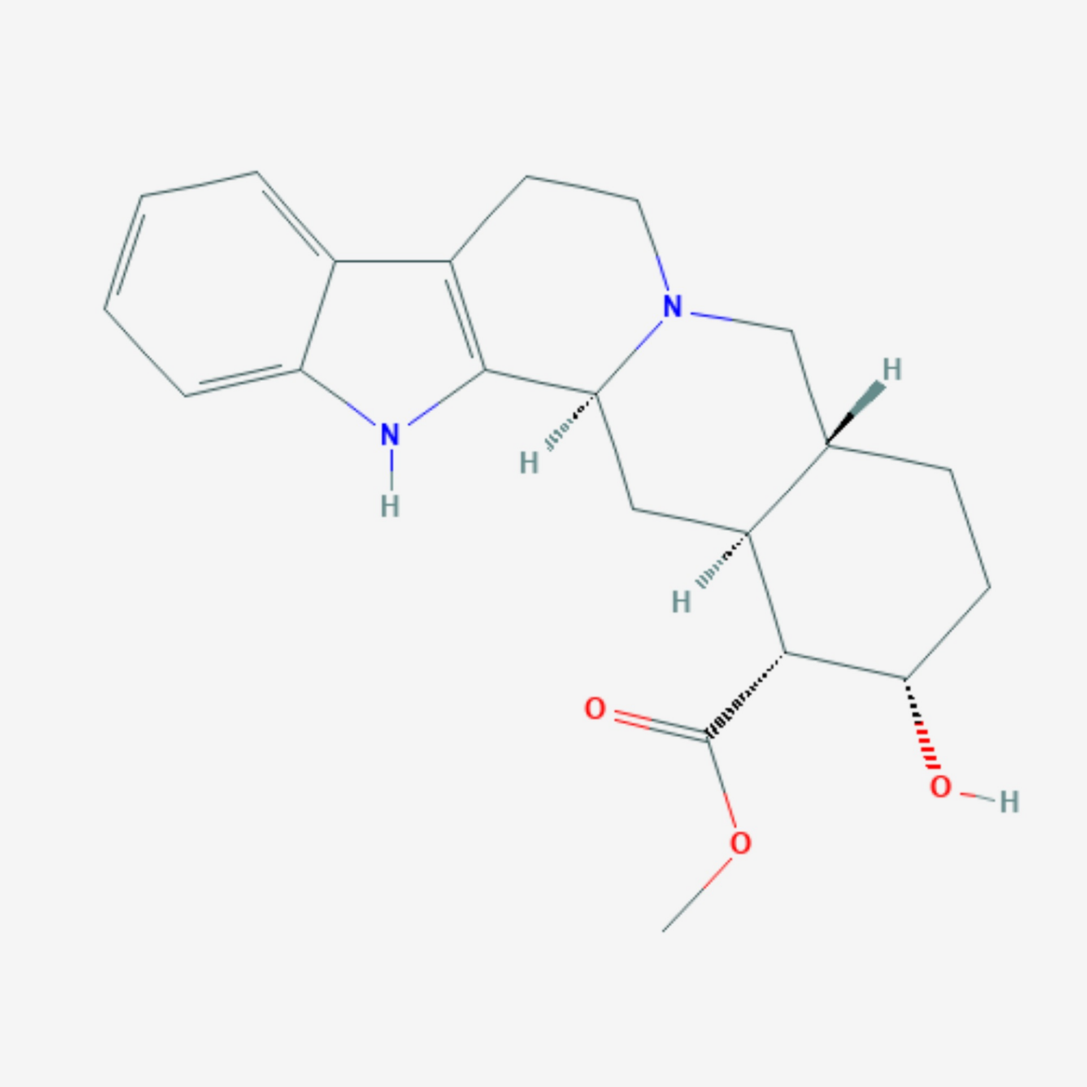
Yohimbine – molecular formula: C21H26N2O3; molecular weight: 354.4 g/mol.

**Figure 5:**
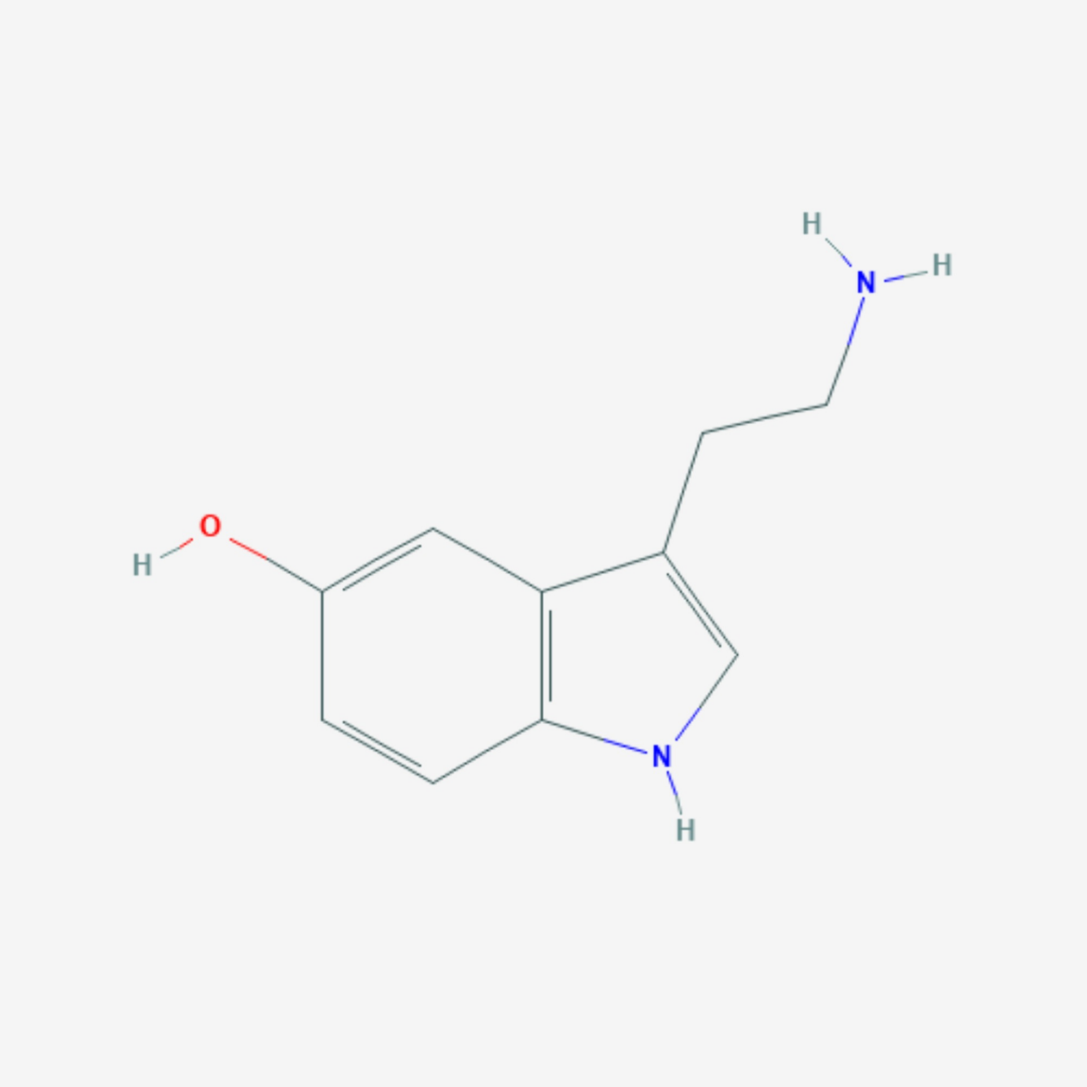
Serotonin (5-hydroxytryptamine) – molecular formula: C10H12N2O; molecular weight: 176.21 g/mol. .

More than 20 active compounds have been isolated from kratom. Mitragynine and 7-hydroxymitragynine are the main psychoactive alkaloids found only in the leaves of Mitragyna speciosa [1,15]. They are selective and full agonists of the mu-subtype opioid receptor (MOR). This agonist effect is antagonized by the opioid receptor antagonist, naloxone. Some studies have reported activity at the delta-subtype opioid receptor as well [12]. Other pharmacological mechanisms through which mitragynine mediates its psychoactive effects include 5-HT2a (5-hydroxytryptamine 2A) and postsynaptic alpha-2 adrenergic receptor activity, as well as activity at neuronal Ca2+ channels. These involve the activation of descending noradrenergic and serotonergic pathways in the spinal cord [12]. Three additional mitragynine analogs with psychoactive properties have been described: speciogynine, paynantheine, and speciociliatine [2]. Mitragynine (Figure 2), 7-hydroxymitragynine (Figure 3), and their analogs contain the bicyclic indole ring and are structurally similar to yohimbine (Figure 4), an indole alkaloid with alpha-2 adrenergic blocking activity as well as serotonergic and dopamine receptor D2 antagonist activity [1,15]. The serotonergic activity of mitragynine, its analogs, and yohimbine can be envisaged considering the similarities in their chemical structure with the endogenous bicyclic indolamine, serotonin (Figure 5) [16-17].

It is interesting to note that yohimbine has been used as a mydriatic to treat erectile dysfunction, is considered an aphrodisiac, and has also been used by elite athletes in fat-loss programs [18]. These properties are similar to those produced by kratom products.

7-hydroxymitragynine has been reported as the more potent alkaloid compared with mitragynine. Although kratom contains more mitragynine, 7-hydroxymitragynine appears to be the predominant mediator of its analgesic effects. 7-hydroxymitragynine has better oral bioavailability and blood-brain barrier penetration than mitragynine. It has been reported that individuals who use kratom for analgesia prefer the stimulant effects of kratom to the sedative effects of opioids. Additionally, although euphoria from kratom tends to be less intense than opioid-induced euphoria, it is still sought by individuals with substance use disorders, reflecting its neurobehavioral effect following chronic use.

Individual characteristics play a role in what a user might experience from kratom use. This experience also depends on the duration of use and the dosage (Tables 1-4). The index case presented with multiple risk factors for adverse effects from kratom use. Kratom intoxication can lead to psychosis and respiratory depression (Table 3). Kratom toxicity can lead to seizures (Table 4), as depicted in the index case. This risk likely became heightened with chronic use as the patient had reported daily kratom use for 1.5 years before his presentation.

**Table 3:** Kratom intoxication

Effects of kratom intoxication
Euphoria, psychosis (hallucinations), mania, agitation, respiratory depression

**Table 4:** Kratom toxicity

Effects of kratom toxicity
Psychosis (hallucinations), mania, agitation, seizures, hypothyroidism, intrahepatic cholestatic injury, sudden cardiac death

The patient’s history of ADHD also suggested a susceptibility to neuropsychiatric sequelae from kratom use as this condition is characterized by problems with dopamine and norepinephrine neurotransmitter activity at cellular and circuitry levels. In ADHD, imaging studies indicate that there are alterations in the prefrontal cortex (PFC) and its connections with the striatum and the cerebellum [19]. The PFC requires optimal levels of norepinephrine and dopamine to function appropriately in controlling behavior and attention. Findings from animal studies suggest that norepinephrine’s activity at postsynaptic alpha 2A receptors in the PFC as well as dopamine’s moderate D1 receptor stimulation work in consonance to mediate these functions. Animal models point to the dysregulation of PFC circuits as the pathophysiology underlying ADHD [19]. While it is possible that mitragynine’s stimulating postsynaptic alpha 2 adrenoceptor activity could theoretically have been beneficial for the patient with regard to ADHD symptoms, consuming high doses daily would have likely canceled out any potential benefits. The patient’s pattern of use and the resulting complication are more reflective of dependence rather than judicious use for clinically justifiable reasons. Furthermore, this patient presented with an impairment in reward processing evidenced by his comorbid benzodiazepine use disorder and opioid use disorder history. His risk of developing an addiction to kratom was therefore elevated. It is also noteworthy to emphasize the impact of the lost health insurance coverage on this patient’s history-the sentinel event that precipitated chronic kratom use and the clinical picture that subsequently emerged.

A review on kratom’s abuse potential has concluded that a complete ban on kratom products could lead to public health concerns including the risk of illicit opioid use and overdose in kratom users [20]. Kratom use in the US is rising, leading to kratom dependence and several medical complications. Kratom use has been linked to hepatotoxicity and several neurological complications including posterior reversible encephalopathy syndrome and seizures, both of which can be life-threatening [6]. Tatum et al. reported that kratom use may be related to structural magnetic resonance imaging (MRI) changes in the brain and that its chronic use may result in recurrent seizures requiring treatment with antiepileptic medications [5]. In our study, MRI brain was not performed as the patient presented with a single seizure episode; however, a case of recurrent seizures following kratom use should prompt MRI studies to evaluate for structural brain changes. Future research should target the effects of kratom on the anatomy and physiology of the central nervous system. 

## Conclusions

Kratom use is on the rise in the US and there is evidence of its potential to cause opioid-type dependence when used chronically. Apart from its abuse potential, kratom use is also associated with serious medical consequences including liver injury, respiratory depression, psychosis, seizures, and other neuropsychiatric complications. We hope that this case report will increase awareness in the medical community on the risk of neuropsychiatric sequelae, particularly seizures, with chronic use of kratom. Kratom's relatively unknown safety profile and easy availability necessitate further research to hopefully provide additional insight into its appropriate regulatory control.
